# Supplementary material for: KCNE1 tunes the sensitivity of KV7.1 to polyunsaturated fatty acids by moving turret residues close to the binding site
Source: eLife. 2018 Jul 17;7:e37257. doi: 10.7554/eLife.37257 (PMC6080945; doi:10.7554/eLife.37257)
Supplement: Supplementary file 1. [file elife-37257-supp1.docx]

| **Supplementary File 1. Summary of biophysical properties of K_V_7.1 and KCNE1 mutants.** | | | |
| --- | --- | --- | --- |
| **Construct** | ***V*_50_**  **pH 7.4 (mV)** | **s**  **pH 7.4 (mV)** | **n** |
| 7.1 (WT) | −29.4 ± 1.4 | 9.3 ± 0.4 | 6 |
| 7.1+E1 (WT) | +20.1 ± 3.2 | 14.0 ± 1.2 | 5 |
|  |  |  |  |
| 7.1+E1/∆N2-38 | +77.0 ± 5.1 | 24.7 ± 2.0 | 4 |
| 7.1+E1/D39C/E43C | +42.9 ± 1.9 | 20.8 ± 0.8 | 6 |
| 7.1+E1/K41C | +46.6 ± 1.4 | 19.5 ± 0.2 | 4 |
| 7.1/E284C+E1 | +27.6 ± 1.8 | 18.1 ± 1.0 | 5 |
| 7.1/D286C+E1 | +28.1 ± 2.6 | 17.4 ± 0.4 | 5 |
| 7.1/E290C+E1 | +26.3 ± 2.8 | 16.5 ± 0.4 | 8 |
| 7.1/E290A+E1 | +27.0 ± 3.9 | 15.7 ± 2.4 | 5 |
| 7.1/E290R+E1 | +21.8 ± 3.3 | 13.2 ± 0.6 | 4 |
| 7.1/E295C+E1 | +28.6 ± 2.7 | 16.3 ± 1.1 | 7 |
| 7.1/D301C+E1 | +18.1 ± 2.4 | 18.6 ± 0.6 | 4 |
|  |  |  |  |
| 7.1/E290C | −28.6 ± 1.4 | 10.6 ± 0.3 | 7 |
|  |  |  |  |
| E1 WT only | +12.1 ± 2.5 | 15.2 ± 0.5 | 4 |
| *V*_50_ and s were determined using Eq. 1 as described in the Methods section. Data shown as mean ± SEM. | | | |
